# Glycaemic Profiles of Children With Overweight and Obesity in Free-living Conditions in Association With Cardiometabolic Risk

**DOI:** 10.1038/srep31892

**Published:** 2016-08-18

**Authors:** Jesse Rijks, Kylie Karnebeek, Jan-Willem van Dijk, Elke Dorenbos, Willem-Jan Gerver, Pauline Stouthart, Jogchum Plat, Anita Vreugdenhil

**Affiliations:** 1 Centre for Overweight Adolescent and Children’s Healthcare, Department of Paediatrics, Maastricht University Medical Centre, Maastricht, The Netherlands; 2 School of Nutrition and Translational Research in Metabolism (NUTRIM), Maastricht University, Maastricht, The Netherlands; 3 Institute of Sports and Exercise Studies, HAN University of Applied Sciences, Nijmegen, The Netherlands; 4 Department of Human Biology, Maastricht University, Maastricht, The Netherlands

## Abstract

Insulin resistance is common among children with overweight and obesity. However, knowledge about glucose fluctuations in these children is scarce. This study aims to evaluate glycaemic profiles in children with overweight and obesity in free-living conditions, and to examine the association between glycaemic profiles with insulin resistance and cardiovascular risk parameters. One hundred eleven children with overweight and obesity were included. 48-hour sensor glucose concentrations in free-living conditions, fasting plasma and post-glucose load concentrations, serum lipid and lipoprotein concentrations, homeostatic model assessment of insulin resistance (HOMA-IR), and blood pressure were evaluated. Hyperglycaemic glucose excursions (≥7.8 mmol/L) were observed in 25% (n = 28) of the children. The median sensor glucose concentration was 5.0 (2.7–7.3) mmol/L, and correlated with fasting plasma glucose concentrations (r_s_ = 0.190, p = 0.046), serum insulin concentrations (r_s_ = 0.218, p = 0.021), and HOMA-IR (r_s_ = 0.230, p = 0.015). The hyperglycaemic area under the curve (AUC) correlated with waist circumference z-score (r_s_ = 0.455, p = 0.025), triacylglycerol concentrations (r_s_ = 0.425, p = 0.024), and HOMA-IR (r_s_ = 0.616, p < 0.001). In conclusion, hyperglycaemic glucose excursions are frequently observed in children with overweight and obesity in free-living conditions. Children with insulin resistance had higher median sensor glucose concentrations and a larger hyperglycaemic sensor glucose AUC, which are both associated with specific parameters predicting cardiovascular disease risk.

Glycaemic dysregulation is an important risk factor for the development of cardiovascular disease^1^,^2^,^3^. Multiple acute hyperglycaemic glucose fluctuations over the day appear more harmful for vasculature than a sustained chronic hyperglycaemic state^4^,^5^. The exact mechanism is not completely understood, but previous studies demonstrated that pathways involved in oxidative stress generation are more activated in response to intermittent glucose fluctuations compared to sustained high glucose concentrations^6^,^7^. It has been hypothesized that very early glycaemic dysregulation, long before the actual onset of type 2 diabetes mellitus (T2DM), already contributes substantially to endothelial and vascular dysfunction^8^,^9^. In keeping with this, in a substantial number of obese adolescents diagnosed with T2DM, serious vascular comorbidities including hypertension, dyslipidaemia, and micro albuminuria were present during the early onset of the disease^10^.

A large number of studies have shown that insulin resistance is already present in a significant number of children with overweight and obesity^11^,^12^. Information on glucose profiles and the effect of insulin sensitivity on these profiles is, however, lacking. In addition, the relevance of glucose fluctuations, especially in the context of future cardiovascular risk, so far remains unknown. In clinical practice alterations in glucose metabolism are usually detected with an oral glucose tolerance test (OGTT). With the OGTT it is possible to detect significant glucose disturbances, however the ability to detect subtle disturbances in glucose homeostasis is limited with this test. Further, the reproducibility of the OGTT in children with metabolic derangements is poor, in particular the 2-hour plasma glucose concentrations^13^. The inconsistent finding specifically in this population might be due to changeable β-cell responses and peripheral insulin sensitivity or other unknown factors. With a continuous glucose monitoring (CGM) sensor it is possible to acquire a detailed insight of glucose fluctuations in the interstitial fluid, which correlates with capillary measurements^14^. Currently, CGM is commonly used in children and adults with diabetes to detect hypo- and hyperglycaemic glucose excursions, with the aim to improve the diabetic regulation through the adjustment of therapy^15^,^16^. So far, studies using CGM to visualize glycaemic profiles in children with overweight and obesity without diabetes in free-living conditions are limited. A recent study in obese adolescents reported that overall glucose concentrations measured in free-living conditions were higher than in a normal weight, healthy control group, despite having normal HbA1c concentrations, fasting glucose concentrations, and 2-hour plasma glucose concentrations after a glucose load^17^,^18^. Whether these disturbances in glucose homeostasis are associated with cardiovascular risk is unclear. Therefore, in this study we evaluated glycaemic profiles using a CGM sensor in children with overweight and obesity in free-living conditions, and examined the association between glycaemic profiles with insulin resistance and cardiovascular risk parameters.

## Results

### Baseline characteristics

One hundred and eleven children (40 boys and 71 girls), predominantly Caucasian (94%), with a mean age of 12.6 ± 3.0 (mean ± standard deviation) years were enrolled in this study. Baseline characteristics are presented in Table 1. Nineteen percent (%) (n = 21) were overweight, 40% (n = 44) obese, and 41% (n = 46) morbidly obese. Mean body mass index (BMI) z-score was 3.42 ± 0.70. Fasting glucose concentrations were normal (<5.6 mmol/L) in all children. Four children (4%) were classified as impaired glucose tolerant (IGT) with plasma glucose concentrations ≥7.8 mmol/L 2-hours after the glucose load. However, none of the children had plasma glucose concentrations ≥11.1 mmol/L 2-hours after the glucose load. In 20% of the children (n = 27) HbA1c concentrations were elevated (≥5.7%). The median homeostatic model assessment of insulin resistance (HOMA-IR) was 2.75 (0.43–14.79) (median with range), and based on the HOMA-IR, insulin resistance was present in 57% (n = 63) of the children.

### 48-hour glycaemic profiles and subgroup analysis

**Figure 1:**
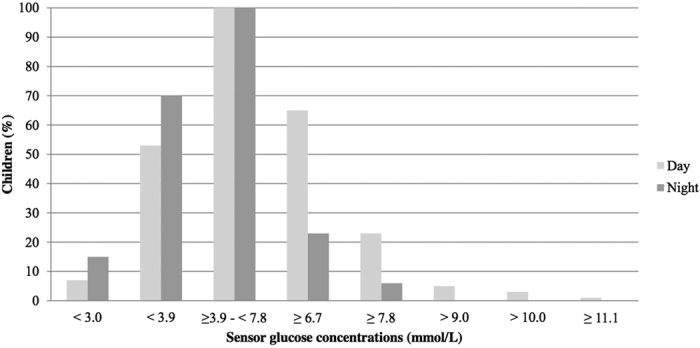
Percentage of children reaching sensor glucose concentrations at any time during the 48 h measurement period. Day = 07:00 am–10:00 pm. Night = 10:00 pm–07:00 am.

**Figure 2:**
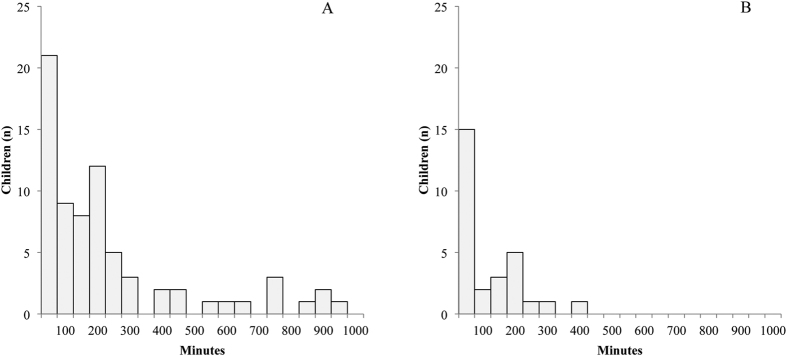
Number of children reaching high-normal and hyperglycaemic sensor glucose concentrations during specified time intervals. (**A**) Duration in minutes sensor glucose concentrations ≥6.7 mmol/L (n = 72; 65% of the total group) (**B**). Duration in minutes sensor glucose concentrations ≥7.8 mmol/L (n = 28; 25% of the total group).

The median 48-hour sensor glucose concentration was 5.0 (2.7–7.3) mmol/L, and was higher during daytime as compared to nighttime. The proportions of children exceeding specific blood glucose concentration thresholds at any time during the CGM period - stratified by day and night - are shown in Fig. 1. Sixty-five percent (n = 72) of the children showed high normal sensor glucose concentrations (≥6.7 mmol/L), for 7.4% of the total time (Fig. 2). Twenty five percent (n = 28) reached hyperglycaemic sensor glucose concentrations (≥7.8 mmol/L), on average 3.3% of the total time (Fig. 2). Anthropometrics and cardiovascular risk parameters did not differ between the children with and without hyperglycaemic sensor glucose concentrations. The duration spent above the hyperglycaemic threshold of 7.8 mmol/L was significantly longer in the insulin resistant children (15 minutes vs. 105 minutes, p = 0.004; Table 2). Seven children exceeded sensor glucose concentrations of 9.0 mmol/L, while 3 children surpassed glucose concentrations of 10.0 mmol/L. The subgroup of children exceeding glucose concentrations of 9.0 mmol/L was too small to perform further statistical analysis. Only one of the children that exceeded sensor glucose concentrations of 9.0 mmol/L was also classified as IGT based on the OGTT. One child reached sensor blood glucose concentrations ≥11.1 mmol/L for 15 minutes, but was not classified as IGT. Seventy percent (n = 78) of the children reached sensor blood glucose concentrations below 3.9 mmol/L, approximately 10.1% of the total time. Generally, these hypoglycaemic sensor glucose concentrations were reached during the night. There were no significant differences between the children with overweight, obesity and morbid obesity in regard to all CGM sensor parameters.

Children with insulin resistance, defined as a HOMA-IR ≥2.5, showed significantly higher median sensor glucose concentrations (p = 0.026) and hyperglycaemic sensor glucose areas under the curve (AUC) (p = 0.003), as compared to those with a HOMA-IR <2.5 (Table 1). Children with insulin resistance also had significantly higher serum triacylglycerol (TAG) concentrations (p = 0.006), a higher diastolic blood pressure (BP) z-score (p = 0.011), and lower serum HDL-cholesterol concentrations (p < 0.001) (Table 1).

Further, when children were stratified by the presence of dyslipidaemia based on serum TAG concentrations or HDL-cholesterol concentrations, HOMA-IR and insulin concentrations were higher in the children with high serum TAG and low serum HDL cholesterol concentrations. Moreover, fasting plasma glucose concentrations, plasma glucose concentrations 2-hours after the glucose load, and all CGM sensor parameters did not differ significantly between the groups.

### Correlations

The 48-hour median sensor glucose concentrations correlated significantly with fasting plasma glucose concentrations (r_s_ = 0.190, p = 0.046), fasting serum insulin concentrations (r_s_ = 0.218, p = 0.021), and HOMA-IR (r_s_ = 0.230, p = 0.015). Interestingly, no significant correlations were found between BMI z-scores and the sensor blood glucose concentrations, or CONGA. Positive correlations were also found between CONGA with HbA1c and TAG concentrations (Table 3).

Within the subgroup of children who reached hyperglycaemic sensor glucose concentrations (n = 28), waist circumference z-scores (r_s_ = 0.455, p = 0.025), fasting serum insulin concentrations (r_s_ = 0.607, p < 0.001), serum TAG concentrations (r_s_ = 0.425, p = 0.024), and HOMA-IR (r_s_ = 0.616, p < 0.001) correlated significantly with the hyperglycaemic sensor glucose AUC. The hypoglycaemic sensor glucose AUC was not associated with BMI z-score, HOMA-IR, or other cardiovascular risk parameters.

## Discussion

Insulin resistance is common among children with overweight and obesity. There is, however, not much known about the occurrence of glucose fluctuations in these children and whether early glucose disturbances are associated with cardiovascular risk. By evaluating glycaemic profiles in children with overweight and obesity in free-living conditions, this study demonstrated that children with insulin resistance have higher median sensor glucose concentrations and a larger hyperglycaemic sensor glucose AUC as compared to children without insulin resistance. Most importantly, median sensor glucose concentrations are associated with plasma fasting glucose concentrations, serum fasting insulin concentrations and HOMA-IR, and the hyperglycaemic sensor glucose AUC is associated with systolic BP z score and serum TAG concentrations. No associations are demonstrated with other lipid or lipoprotein concentrations.

Although median sensor glucose concentrations appeared to be within normal range, short-term hyperglycaemic excursions were frequently observed in children with overweight and obesity in free-living conditions. Little information is available about the occurrence of hyperglycaemic excursions in normal weight, healthy children. In the one study that investigated glucose concentrations in free-living conditions in normal weight, healthy children hyperglycaemic excursions were rarely found, in contrast to children with overweight and obesity studied in our study. The percentage of time spent above the hyperglycaemic threshold of 7.8 mmol/L by the children in our study, is in line with the percentage of time spent by obese adolescents without prediabetes (0.5% vs. 1.3%), as reported by Chan *et al.*^17^.

From a clinical perspective, it is important to obtain more knowledge about the relevance of hyperglycaemic glucose excursions in free-living conditions in children with overweight and obesity. In adults with T2DM, hyperglycaemia causes endothelial dysfunction and contributes to vascular damage^19^,^20^. It is plausible that the same harmful mechanisms affect the vascular system during childhood hyperglycaemia. However, in children with overweight and obesity it is unknown if hyperglycaemic glucose concentrations are already harmful. Although this study showed that there were no significant differences in cardiovascular risk parameters between children with and without hyperglycaemic glucose concentrations, the hyperglycaemic sensor glucose AUC revealed a modest but positive correlation with several cardiovascular risks parameters. This suggests that simply the presence or absence of hyperglycaemic glucose concentrations is not determinative for the initiation of cardiovascular derangements. Instead it seems that duration and frequency of hyperglycaemic glucose concentrations are defining the association with cardiovascular risk parameters. Interestingly, hyperglycaemic sensor glucose AUC correlated specifically with waist circumference z-score, TAG concentrations, and HOMA-IR, but not with the other cardiovascular risk parameters investigated in this study.

Daytime median glucose concentrations in free-living conditions were positively correlated with serum TAG concentrations and systolic BP. Also, significant correlations were found between CONGA and serum TAG concentrations. These results signify that subtle elevations of glucose concentrations, greater glycaemic variability, and the AUC of hyperglycaemic glucose concentrations are associated with specific cardiovascular risk parameters in children with overweight and obesity. Further, the majority of the children (73%) reached hypoglycaemic sensor glucose concentrations, which is uncommon in normal weight, healthy children^18^. Interestingly, children who reached hypo- or hyperglycaemic sensor glucose concentrations could not be identified using the measurements generally used in clinical practice (e.g. fasting glucose and insulin concentrations, HOMA-IR, glucose concentrations 2-hours after a glucose load). Median sensor glucose concentrations only illustrated a weak correlation with commonly used measurements such as fasting plasma glucose concentrations, serum insulin concentrations, and HOMA-IR. In addition, this study showed that glucose concentrations in free-living conditions are not simply the consequence of excess body weight, since no associations were found between BMI z-score and CGM sensor parameters. Other factors such a lifestyle factors, pro-inflammatory status, and beta cell functioning might be involved in glucose concentrations in free-living conditions.

Higher CONGA values indicate a greater glycaemic variation^21^. Nevertheless, reference values for normal weight, healthy children are unknown. While CONGA reflects intra-day glycaemic variability, HbA1c reflects the glycaemic control over a three-month period. Daytime CONGA values correlated positively with HbA1c concentrations in this study population. Since daytime glycaemic variability is probably influenced by dietary habits and physical activity, it is tempting to suggest that post-prandial glucose excursions affect HbA1c concentrations, which is in line with previous findings in adults with T2DM^22^,^23^.

So far, insulin resistance has not been studied in the context of glycaemic profiles of children with overweight and obesity in free-living conditions. This is the first study demonstrating that children with insulin resistance had significantly higher glucose concentrations in free-living conditions, as compared to children without insulin resistance. Furthermore, the hyperglycaemic sensor glucose AUC was significantly larger in children with insulin resistance. As described above, subtle glucose elevations as well as increased hyperglycaemic sensor glucose AUC are associated with increased cardiovascular risk. Children with insulin resistance showed more worrisome cardiovascular risk profiles in contrast to children without insulin resistance, as evidenced by significantly greater waist circumferences, higher serum TAG concentrations, higher HbA1c concentrations, lower serum HDL-cholesterol, and higher diastolic BP. These new data provide valuable information for hypothesises about the associations between glucose dysregulation and cardiovascular risk markers. Since our results illustrated that dyslipidaemia appears in the absence of severe glucose excursions, we hypothesize that either high serum TAG concentrations and/or low HDL-cholesterol concentrations are involved in the transition from insulin resistance to hyperglycaemia, or even that dyslipidaemia is causal to the development of insulin resistance. However, the possibility that dyslipidaemia and glucose dysregulation occur both as a separate response to the excess in weight, depending on individual susceptibility, should also be considered. Longitudinal studies are necessary to unravel the exact sequence of events eventually leading to glucose dysregulation.

There are several limitations that should be considered when interpreting the results of this study. Even though the children were asked to maintain their own eating and exercise habits, it is possible that they have shown restrictive behaviour, which directly reflects on glucose concentrations. Therefore the presence and duration of hyperglycaemic excursions, and the degree of glycaemic variability might be underestimated. Further, the CGM sensor measurement was only executed once. More frequent measurements over a longer period of time would increase the reliability of the findings. Since studies investigating glycaemic profiles in non-diabetic children with overweight and obesity in free-living conditions are scarce, affirmation of our findings in other cohorts is recommendable. It would also have been valuable if normal weight, healthy children were included in this study, considering that evidence regarding glucose concentrations in free-living conditions in this population is limited to only one study. In this study we evaluated associations of CGM data with established cardiovascular risk markers, such as blood pressure and biochemical markers in plasma. To gain more insight into the associations of glucose homeostasis and early vascular deterioration it could also be valuable to relate to early markers of macro- and microvascular function (e.g. pro-inflammatory cytokines, endothelial adhesion molecules, retinal vascular diameters, pulse wave velocity). Notably, HOMA-IR was used in this study to assess insulin resistance, while the euglycaemic hyperinsulemic clamp technique is considered to be the gold standard. However, this is not easily applicable in a clinical setting and especially challenging to perform in children. HOMA-IR is a simple, inexpensive substitute for insulin resistance derived from a mathematical assessment of the balance between hepatic glucose output and insulin secretion, for which only fasting plasma glucose and fasting serum insulin are required^24^. It is considered to be a valid tool in assessing insulin resistance in children with obesity^25^.

In conclusion, hyperglycaemic glucose fluctuations are frequently present in children with overweight and obesity in free-living conditions. Children with insulin resistance have significantly higher median sensor glucose concentrations and a larger hyperglycaemic sensor glucose AUC, this is both associated with increased cardiovascular disease risk. Long-term longitudinal follow-up studies in a large population are necessary to investigate whether glycaemic profiles can provide early identification of children at high risk for developing T2DM and cardiovascular diseases. As well, it can be valuable to investigate the influence of lifestyle improvement on glucose concentrations in free-living conditions.

## Methods

### Setting

This study was designed and conducted within the setting of the Centre for Overweight Adolescent and Children’s Healthcare (COACH) at the Maastricht University Medical Centre (MUMC+). Within COACH, the health status of children with overweight and obesity, and their families was evaluated, and they were monitored and received lifestyle coaching as described previously^26^. Briefly, participation in the COACH program commenced with a comprehensive assessment aimed to exclude underlying syndromic or endocrine conditions of overweight, evaluate complications and risk factors, and obtain insight into behaviour and family functioning. The assessment included, amongst others, an OGTT and a CGM sensor measurement. After the assessment, all children and their families were offered on-going, tailored and individual guidance with foci on lifestyle changes on a frequent basis at the outpatient clinic.

### Study participants

All 168 children who started participating in the COACH program between 2011–2013 and who received a CGM measurement were considered for inclusion in this study. Children with incomplete CGM sensor data or in whom the software failed to extract the data (n = 42), with diabetes mellitus (n = 1), and missing fasting plasma glucose or serum insulin concentrations (n = 14) were excluded from this study. Finally, 111 children were eligible for inclusion. The study was conducted according to the guidelines administered by the Declaration of Helsinki and approved by the medical ethical committee of the MUMC+. Informed consent was subsequently obtained.

### Participant characteristics

Anthropometric data was acquired while children were barefoot and wearing only underwear. Body weight was determined using a digital scale (Seca) and body length was measured using a digital stadiometer (De Grood Metaaltechniek). BMI was calculated and BMI z-scores were obtained using a growth analyser (Growth Analyser VE) based upon reference charts of the Dutch nationwide growth study^27^. Based on the International Obesity Task Force criteria children were classified as overweight, obese, or morbidly obese^28^. Waist circumference was measured with a non-elastic tape at the end of a natural breath at midpoint between the top of the iliac crest and the lower margin of the last palpable rib. Waist circumference z-scores were calculated according to age references for Dutch children^29^. Ethnicity was defined based on the definition of the Dutch Central Agency for Statistics^30^.

### Glucose metabolism

After obtaining the fasting blood sample an OGTT was performed. 1.75 grams of glucose per kilogram of bodyweight was dissolved into 200 mL water, with a maximum of 75 grams of glucose in total, and given orally. Plasma blood glucose concentrations were measured every thirty minutes during two hours. Impaired fasting glucose (IFG; fasting glucose 5.6–6.9 mmol/L), IGT (glucose ≥7.8–<11.1 mmol/L after 2-hours), T2DM (fasting glucose ≥7.0 mmol/L or glucose ≥11.1 mmol/L after 2-hours), and elevated HbA1c concentrations (≥5.7%) were classified according to the American Diabetes Association (ADA) criteria^31^. In this study, insulin resistance was estimated using the HOMA-IR^24^. The following formula was applied: fasting glucose (mmol/L) × fasting insulin (μU/L)/22.5^24^. A cut-off point of 2.5 was used exercised based on adult standards to determine the presence of insulin resistance^24^.

In addition to the OGTT data and HOMA-IR, CGM sensors were used to determine glucose concentrations in free-living conditions. Dependent on the preference of the child the CGM sensor (Medtronic) was inserted subcutaneously in the upper leg or arm in the morning in the hospital. Interstitial fluid glucose levels were measured every five minutes with the CGM sensor for 72-hours. To ensure all sensor blood glucose measurements were obtained in free-living conditions the values of the second and third day (both from 00:00–23:59) were used for analysis (48-hour in total). Calibration of the device required capillary glucose readings three times per day. This was obtained through self-monitored capillary glucose samples: fasting, in the afternoon and pre-bedtime using the Accu-Chek (Roche). All children were asked to maintain habitual eating and physical activity patterns. After 72-hours, the sensor was removed, and the data was uploaded to an online software program (Medtronic Carelink-software), and subsequently downloaded.

Afterwards, 48-hour sensor blood glucose concentrations were calculated. Median sensor glucose concentrations, and the prevalence of hypoglycaemia (<3.9 mmol/L)^32^ and hyperglycaemia (≥7.8 mmol/L)^31^ were calculated. In addition, the International Diabetes Federation criteria for maximum postprandial glucose concentration (>9.0 mmol/L)^33^ and the maximum postprandial glucose concentration (>10.0 mmol/L)^31^ according to the ADA criteria were used. Since glucose fluctuations are influenced by dietary habits and physical activity during the day, daytime (07.00 am–10.00 pm) and nighttime (10.00 pm–7.00 am) sensor glucose concentrations were also evaluated separately. The timeframe for day and night was based on the mean self-reported sleeping time. The intra-day glycaemic variability, which reflects acute glucose fluctuations, was assessed by the CONGA. With this method, the difference between each glucose reading and the glucose reading n hours previously is calculated^21^. The CONGA is the standard deviation of the differences. In this study, CONGA1, CONGA2, and CONGA4 were used based on 1-, 2- and 4-hour time differences, respectively. In essence, the time differences corresponded approximately to time between different activities in school, time between snacks, and time between meals^21^.

Total OGTT AUC and total sensor glucose AUC were calculated using the trapezoidal method. The AUC is an integrated measurement reflecting the duration and magnitude of the glucose concentrations. Hypo- and hyperglycaemic sensor glucose AUC were also calculated, reflecting the AUC for sensor glucose concentrations <3.9 mmol/L and ≥7.8 mmol/L respectively.

### Cardiovascular risk

Fasting lipid profiles, including serum total cholesterol, LDL-cholesterol, HDL-cholesterol, and TAG concentrations, were measured. Daytime BP was measured during a period of 1.5 hours for approximately 20 times with an interval of three minutes between each measurement using the Mobil-O-Graph (I.E.M. GmbH). Mean BP was calculated. The size of the cuff used corresponded with the circumference of the upper arm. Systolic- and diastolic BP z-scores were calculated according to reference values related to height and gender^34^.

### Biochemical analysis

Fasting plasma glucose concentrations and serum total cholesterol, LDL-cholesterol, HDL-cholesterol, and TAG concentrations were determined with the Cobas 8000 modular analyser (Roche). Serum insulin concentrations were analysed with the Immulite-1000 (Siemens Healthcare Diagnostics). HbA1c concentrations were determined with the fully automated HPLC Variant II (Bio-Rad Laboratories).

### Statistical analysis

All statistical analyses were performed using SPSS 20.0 for Windows (SPSS Inc, Chicago, IL). Differences in baseline characteristics between groups were analysed with a X^2^-test, Student’s T-test, or Mann-Whitney U-test, as appropriate. Correlations between variables were determined by Spearman’s correlation analysis. Data are presented as means with standard deviations or as medians with ranges. For all analysis, a p-value below 0.05 was considered to be statistically significant.

### Clinical Trail registration

ClinicalTrial.gov; *Registration Number:* NCT02091544.

## Additional Information

**How to cite this article**: Rijks, J. *et al.* Glycaemic Profiles of Children With Overweight and Obesity in Free-living Conditions in Association With Cardiometabolic Risk. *Sci. Rep.* **6**, 31892; doi: 10.1038/srep31892 (2016).

## Figures and Tables

**Table 1:** Characteristics of the study participants stratified by insulin resistance.

	Total (n = 111)	HOMA-IR <2.5 (n = 48)	HOMA-IR ≥2.5 (n = 63)
*Age*	12.5 ± 3.0	12.1 ± 3.3	12.8 ± 2.7
*Male/Female, %*	36 /64	42/58	32/68
*Caucasian*^a^*, %*	94	94	94
*Positive family history of diabetes*^b^, *%*	68	64	71
*BMI z-score*	3.42 ± 0.70	3.29 ± 0.68	3.53 ± 0.71
*Overweight/ obese/ morbidly obese*^c^*, %*	19/40/41	25/46/ 29	14/35/51
*Waist circumference z-score*	5.4 (1.4–13.9)	4.4 (1.4–11.9)^f^	6.7 (2.9–13.9)^f^
*Glucose, mmol/L*	4.1 (2.1–5.2)	4.0 (2.1–5.1)^e^	4.2 (2.5–5.2)^e^
*Insulin, mU/L*	15.3 (2.4–72.3)	8.9 (2.4–16.7)^f^	20.8 (8.6–72.3)^f^
*HOMA-IR*	2.75 (0.43–14.79)	1.66 (0.43–2.48)^f^	3.97 (2.50–14.79)^f^
*HbA1c, %*	5.4 (3.1–6.2)	5.2 (4.7–5.8)^f^	5.5 (3.1–6.2)^f^
*Plasma glucose 2-hours after glucose load, mmol/L*	5.4 (2.6–9.0)	5.3 (2.7–9.0)	5.7 (2.6–9.0)
*AUC OGTT*	12540 (8189–20351)	12162 (8189–18378)^f^	13230 (8973–20351)^f^
*Total cholesterol, mmol/L*	4.4 ± 0.8	4.4 ± 0.8	4.5 ± 0.8
*LDL-cholesterol, mmol/L*	2.7 ± 0.7	2.6 ± 0.7	2.8 ± 0.7
*HDL-cholesterol, mmol/L*	1.2 ± 0.3	1.3 ± 0.3^f^	1.1 ± 0.3^f^
*Triacylglycerol, mmol/L*	1.16 ± 0.68	0.96 ± 0.52^f^	1.32 ± 0.75^f^
*Systolic blood pressure z-score*	0.23 ± 1.11	0.02 ± 1.08	0.41 ± 1.11
*Diastolic blood pressure z-score*	−0.52 ± 1.09	−0.83 ± 1.07^e^	−0.28 ± 1.04^e^
*Median sensor glucose, mmol/L*	5.0 (2.7–7.3)	4.7 (2.7–6.9)^e^	5.1 (3.6–7.3)^e^
*Day*^d^*, mmol/L*	5.2 (4.0–6.7)	5.2 (4.3–6.4)	5.2 (4.0–6.7)
*Night*^d^*, mmol/L*	5.0 (2.7–7.3)	4.7 (2.7–6.9)^e^	5.1 (3.6–7.3)^e^
*Maximum sensor glucose, mmol/L*	7.0 (4.9–11.2)	6.9 (5.6–11.2)	7.0 (4.9–10.8)
*Day*^d^*, mmol/L*	6.9 (4.6–11.2)	6.8 (5.6–11.2)	7.0 (4.6–10.8)
*Night*^d^*, mmol/L*	6.1 (4.4–8.7)	6.1 (4.4–8.3)	6.1 (4.4–8.7)
*Minimum sensor glucose, mmol/L*	3.4 (2.2–5.1)	3.4 (2.2–5.1)	3.4 (2.2–4.6)
*Day*^d^*, mmol/L*	3.8 (2.3–5.2)	3.7 (2.3–5.2)	3.9 (2.4–4.8)
*Night*^d^*, mmol/L*	3.5 (2.2–5.1)	3.6 (2.2–5.1)	3.5 (2.2–4.8)
*CONGA1*	0.58 (0.28- 1.31)	0.58 (0.28–1.31)	0.59 (0.28–1.28)
*Day*^d^,	0.64 (0.27–1.56)	0.62 (0.34–1.56)	0.64 (0.27–1.55)
*Night*^d^,	0.44 (0.15–0.85)	0.43 (0.17–0.85)	0.44 (0.15–0.81)
*CONGA2*	0.72 (0.31–1.62)	0.72 (0.31–1.61)	0.72 (0.33–1.62)
*Day*^d^,	0.72 (0.30–1.92)	0.72 (0.33–1.92)	0.73 (0.30–1.90)
*Night*^d^,	0.49 (0.16–1.10)	0.52 (0.18–1.10)	0.47 (0.16–1.08)
*CONGA4*	0.85 (0.35–2.06)	0.88 (0.37–2.02)	0.82 (0.35–2.06)
*Day*^d^,	0.78 (0.35–2.31)	0.80 (0.35–1.80)	0.75 (0.35–2.31)
*Night*^d^,	0.51 (0.16–1.24)	0.64 (0.17–1.24)	0.49 (0.16–1.11)
AUC sensor glucose	2.61 × 10^5^ (2.06 × 10^5^–3.22 × 10^5^)	2.60 × 10^5^ (2.06 × 10^5^–3.22 × 10^5^)	2.64 × 10^5^ (2.11 × 10^5^–3.19 × 10^5^)
AUC sensor glucose <3.9*	900 (74–4103)	1063 (110–4103)	652 (74–3238)
AUC sensor glucose ≥7.8**	883 (78–3547)	158 (78–1826)^f^	1039 (157–3547)^f^

Data presented as mean ± SD or as median (minimum-maximum); HOMA-IR = Homeostatic Model Assessment of Insulin Resistance; Insulin resistance = HOMA-IR ≥2.5; OGTT = Oral Glucose Tolerance Test; AUC = Area Under the Curve; CONGA = Continuous Overlapping Net Glycaemic Action; CONGA presented for 1, 2, or 4-hour time differences; *n total = 82, n HOMA-IR <2.5 = 35, n HOMA-IR ≥2.5 = 47; **n total = 28, n HOMA-IR <2.5 = 13, n HOMA-IR ≥2.5 = 15.

^a^According to the Dutch Central Agency for Statistics^30^.

^b^First- or second-degree family member.

^c^According to the International Obesity Taskforce Criteria^28^.

^d^Day = 07:00 am–10:00 pm. Night = 10:00 pm–07:00 am.

^e^Significant difference between the two groups at the 0.05 level.

^f^Significant difference between the two groups at the 0.01 level.

**Table 2:** Reaching glucose thresholds during the 48-hour continues glucose monitoring period stratified by insulin resistance.

		HOMA-IR <2.5 (n = 48)				HOMA-IR ≥2.5 (n = 63)			
		% children (n)	Median time in minutes per 48 h	% of the time^b^	% of the total time (n = 111)^c^	% children (n)	Median time in minutes per 48 h	% of the time^b^	% of the total time (n = 111)^c^
*Sensor glucose* <*3.0*	*Overall*	17% (8)	55 (5–395)	4.6%	0.8%	16% (10)	78 (15–310)	3.5%	0.6%
*mmol/L*	*Day*^a^	8% (4)	28 (5–50)	1.5%	0.1%	6% (4)	55 (15–100)	3.1%	0.2%
	*Night*^a^	17% (8)	55 (45–350)	11.1%	1.9%	14% (9)	60 (15–210)	7.9%	1.1%
*Sensor glucose* <*3.9*	*Overall*	73% (35)	310 (25–1265)	11.5%	8.4%	75% (47)	170 (15–890)	9.1%	6.8%
*mmol/L*	*Day*^a^	58% (28)	75 (15–295)	5.6%	3.3%	49% (31)	55 (5–480)	6.1%	3.0%
	*Night*^a^	71% (34)	228 (25–970)	23.8%	16.9%	70% (44)	142 (5–725)	18.8%	13.2%
*Sensor glucose ≥3.9*–<*7.8*	*Overall*	100% (48)	2693 (1615–2880)	91.2%	91.2%	100% (63)	2755 (1990–2880)	92.1%	92.1%
*mmol/L*	*Day*^a^	100% (48)	1752 (1505–1800)	96.0%	96.0%	100% (63)	1770 (1320–1800)	95.4%	95.4%
	*Night*^a^	100% (48)	965 (110–1080)	83.0%	83.0%	100% (63)	1015 (355–1080)	86.6%	86.6%
*Sensor glucose ≥6.7*	*Overall*	58% (28)	113 (10–875)	6.4%	3.8%	70% (44)	148 (5–925)	8.1%	5.6%
*mmol/L*	*Day*^a^	58% (28)	85 (10–595)	8.3%	4.9%	70% (44)	105 (5–925)	10.9%	7.6%
	*Night*^a^	19% (9)	75 (5–280)	10.2%	1.9%	25% (16)	78 (5–325)	9.3%	2.4%
*Sensor glucose ≥7.8*	*Overall*	27% (13)	15 (5–185)^e^	1.8%	0.5%	24% (15)	105 (15–400)^e^	4.5%	1.1%
*mmol/L*	*Day*^a^	23% (11)	15 (5–185)^d^	3.1%	0.7%	22% (14)	108 (5–390)^d^	7.3%	1.6%
	*Night*^a^	4% (2)	18 (5–30)	1.6%	<0.1%	8% (5)	30 (10–50)	3.1%	0.2%
*Sensor glucose >9.0*	*Overall*	4% (2)	50 (30–70)	1.7%	<0.1%	5% (3)	45 (30–115)	2.2%	0.1%
*mmol/L*	*Day*^a^	4% (2)	50 (30–70)	2.8%	0.1%	5% (3)	45 (30–115)	3.5%	0.2%
	*Night*^a^	0% (0)	0	0%	0%	0% (0)	0	0%	0%
*Sensor glucose >10.0*	*Overall*	2% (1)	40	1.4%	<0.1%	3% (2)	20 (15–25)	0.7%	<0.1%
*mmol/L*	*Day*^a^	2% (1)	40	2.2%	<0.1%	3% (2)	20 (15–25)	1.1%	<0.1%
	*Night*^a^	0% (0)	0	0%	0%	0% (0)	0	0%	0%
*Sensor glucose ≥11.1*	*Overall*	1% (1)	15	0.5%	<0.1%	0% (0)	0	0%	0%
*mmol/L*	*Day*^a^	2% (1)	15	0.8%	<0.1%	0% (0)	0	0%	0%
	*Night*^a^	0% (0)	0	0%	0%	0% (0)	0	0%	0%

Percentage of children reaching certain glucose thresholds at any time during the 48-hour continues glucose monitoring period; Data presented as median (minimum–maximum). HOMA-IR = Homeostatic Model Assessment of Insulin Resistance; Insulin resistance = HOMA-IR ≥2.5.

^a^Day = 07:00 am–10:00 pm. Night = 10:00 pm–07:00 am.

^b^% of the time was calculated for the group of children who reached the certain glucose threshold.

^c^% of the time was calculated for the complete group of children.

^d^Significant difference between the median time in minutes between the two HOMA-IR groups at the 0.05 level.

^e^Significant difference between the median time in minutes between the two HOMA-IR groups at the 0.01 level.

**Table 3:** Correlation coefficients between baseline characteristics and continues glucose monitoring data.

		Age	BMI z-score	Waist- circum ference z-score	Fasting glucose	Fasting insulin	HOMA-IR	Plasma glucose t = 120	AUC OGTT	HbA1c	Total choles-terol	LDL choles-terol	HDL choles-terol	Triacyl-glycerol	Systolic BP z-score	Diastolic BP z-score
*Median sensor glucose*	*Overall*	0.003	0.032	0.107	**0.190**^c^	**0.218**^b^	**0.230**^b^	0.011	0.101	0.126	0.012	0.005	−0.149	0.140	0.048	0.075
	*Day*^a^	−0.068	0.036	0.068	−0.081	0.106	0.047	−0.038	−0.002	0.102	−0.002	−0.114	−0.021	**0.294**^c^	**0.229**^b^	0.185
	*Night*^a^	−0.011	0.032	0.107	**0.190**^c^	**0.218**^b^	**0.230**^b^	0.011	0.101	0.126	0.012	0.005	−0.149	0.140	0.048	0.075
*Maximum sensor glucose*	*Overall*	0.005	0.049	0.197	−0.115	0.173	0.116	0.087	0.120	0.151	−0.032	−0.076	−0.067	**0.275**^c^	0.034	0.053
	*Day*^a^	−0.011	0.045	0.184	−0.121	0.185	0.124	0.109	0.156	0.153	−0.039	−0.091	−0.043	**0.273**^c^	0.039	0.043
	*Night*^a^	0.090	0.112	0.186	−0.025	0.064	0.040	0.068	0.040	**0.194**^b^	−0.068	−0.104	−0.127	0.152	0.048	0.061
*Minimum sensor glucose*	*Overall*	−0.014	0.124	0.076	0.070	0.000	0.023	−0.090	−0.108	0.105	0.030	0.004	−0.074	0.071	0.129	0.117
	*Day*^a^	−0.064	0.082	0.103	0.008	0.049	0.033	−0.028	−0.059	0.055	−0.005	−0.053	−0.047	0.039	0.103	0.043
	*Night*^a^	0.016	0.133	0.076	0.070	0.090	0.111	−0.119	−0.066	0.174	0.076	0.014	−0.115	0.181	**0.266**^c^	**0.196**^b^
*CONGA1*	*Overall*	−0.022	0.107	0.188	−0.085	**0.188**^b^	0.135	0.147	**0.221**^b^	**0.212**^b^	−0.109	−0.16	−0.075	**0.247**^c^	−0.026	−0.036
	*Day*^a^	−0.023	0.083	0.174	−0.074	**0.204**^b^	0.162	0.169	**0.258**^c^	**0.257**^c^	−0.106	−0.148	−0.051	**0.221**^b^	0.01	−0.035
	*Night* a	0.037	0.125	0.156	−0.081	0.049	−0.004	0.089	0.101	0.000	−0.088	−0.112	−0.092	0.157	−0.139	−0.03
*CONGA2*	*Overall*	0.023	0.124	**0.201**^b^	−0.054	0.142	0.098	0.161	**0.254**^c^	0.178	−0.139	−0.18	−0.05	**0.205**^b^	−0.075	−0.071
	*Day*^a^	0.052	0.099	0.191	−0.038	0.176	0.148	0.177	**0.275**^c^	**0.264**^c^	−0.136	−0.176	0.009	0.185	−0.05	−0.058
	*Night*^a^	−0.004	0.125	0.164	−0.101	−0.028	−0.078	−0.018	−0.006	−0.005	−0.142	−0.162	−0.076	0.067	−0.188	−0.023
*CONGA4*	*Overall*	−0.001	0.100	0.188	−0.053	0.081	0.038	0.120	**0.230**^b^	0.113	−0.106	−0.134	−0.051	**0.190**^b^	−0.085	−0.09
	*Day*^a^	0.065	0.144	**0.247**^b^	−0.068	0.172	0.135	0.146	**0.258**^c^	**0.267**^c^	−0.068	−0.098	−0.057	**0.248**^c^	−0.005	−0.036
	*Night*^a^	0.002	0.107	0.149	−0.091	−0.052	−0.098	−0.025	0.013	0.024	−0.129	−0.114	−0.075	0.030	**−0.200**^b^	−0.082
*AUC sensor glucose*	*Overall*	0.027	0.117	0.159	−0.052	0.183	0.133	−0.021	−0.014	0.157	−0.006	−0.120	−0.079	**0.340**^c^	**0.239**^b^	0.161

HOMA-IR = Homeostatic Model Assessment of Insulin Resistance; CONGA = Continuous Overlapping Net Glycaemic Action; CONGA presented for 1, 2, or 4-hour time differences; AUC = Area Under the Curve. Correlations between variables were determined by Spearman’s correlation analysis.

^a^Day = 07:00 am–10:00 pm. Night = 10:00 pm–07:00 am.

^b^Significant correlation at the 0.05 level.

^c^Significant correlation at the 0.01 level.
